# Assessing intrinsic capacity for person‐centred HIV care: a cross‐sectional study in ageing populations in Malaysia and Hong Kong

**DOI:** 10.1002/jia2.26404

**Published:** 2024-12-26

**Authors:** Reena Rajasuriar, Syaza Hisham, John Son Lim, Jean Yi Cheong, Wen Ying Ho, Siew Hwei Yap, Nurul Syuhada Zulhaimi, Malinee Neelamegam, Catherine Cheung, Vivian Wong, Ruhana Che Yusof, Kejal Hasmukharay, Shahrul Bahyah Kamaruzzaman, Sharifah Faridah Syed Omar, Meng Li Chong, Pui Li Wong, Grace Chung‐Yan Lui

**Affiliations:** ^1^ Centre of Excellence for Research in AIDS Universiti Malaya Kuala Lumpur Malaysia; ^2^ Department of Medicine Faculty of Medicine Universiti Malaya Kuala Lumpur Malaysia; ^3^ School of Public Health The University of North Texas Health Science Centre Fort Worth Texas USA; ^4^ Department of Medicine and Therapeutics The Chinese University of Hong Kong Hong Kong Hong Kong

**Keywords:** Asia Pacific, frailty, HIV, intrinsic capacity, person‐centred care, WHO Healthy Ageing

## Abstract

**Introduction:**

WHO's Integrated Care for Older People (ICOPE) proposes we measure the functional construct of intrinsic capacity (IC) to monitor and identify individuals with age‐associated vulnerabilities. Assessments of IC may be useful to address the evolving, non‐HV care needs of ageing people with HIV (PWH). However, to date, its utility within the context of HIV has not been assessed.

**Methods:**

Participants included 200 PWH attending out‐patient care (2021−2023) in Universiti Malaya Medical Centre, Malaysia and 101 community controls aged 35 years and above. The ICOPE framework was adapted to derive aggregate IC scores (ranging 0–6) encompassing the five domains of cognition, sensory (hearing and vision), mobility, mood and vitality. Multivariable analyses were used to explore the association of IC scores in PWH with multiple health outcomes including frailty, difficulties performing instrumental activities of daily living (IADL) and inflammatory markers. Area under the receiver operator characteristic (AUC‐ROC) was calculated to predict frailty and IADL deficits in the current cohort and an independent cohort of 275 PWH from Hong Kong (HK).

**Results:**

Median (interquartile range, IQR) age among PWH and controls were 50 (42−56) and 50 (39−59) years, respectively. There were more males among PWH (83% vs. 56%, *p*<0.001). All PWH received antiretroviral therapy (ART) for a median duration of 11 (8−14) years. Aggregate IC scores were lower in PWH but not significantly different compared to controls, (5.4 vs. 5.6, *p* = 0.093) and PWH performed significantly worse than controls only in the cognitive domain. Aggregate IC scores in PWH was independently associated with frailty (OR 0.17 95% CI 0.07−0.42, *p*<0.001), IADL deficits (OR 0.25 95% CI 0.14−0.46, *p*<0.001) and all other patient‐reported outcomes assessed. Aggregate IC scores correlated with IL‐6 but not sCD14 and sCD163 levels. IC scores performed well in identifying PWH with frailty (AUC‐ROC ≥ 0.80) in the HK and Malaysian cohorts but more modestly (AUC‐ROC ≥ 0.64) for IADL deficits.

**Conclusions:**

IC is a good composite measure to monitor non‐HIV, age‐associated physical and social vulnerabilities in PWH on ART and should complement disease‐based monitoring in routine HIV care. Assessments of IC should be validated in larger, longitudinal cohorts of PWH from diverse settings.

## 1 INTRODUCTION

The life expectancy of people living with HIV (PWH) has increased significantly with antiretroviral therapy (ART) leading to a significant number acquiring age‐related comorbidities, often at an earlier age of onset [1]. Consequently, PWH experience greater disability and poorer quality of life compared to age‐matched individuals without HIV [2]. HIV programmes which have historically focused on monitoring viral load and CD4 T‐cell counts as measures of successful HIV care, are now exploring alternative models of care to better address the health needs of PWH as they grow older [3].

The vast majority of PWH reside in low‐middle income settings where access to specialist care to address issues of ageing are limited [4]. This leads to the lack of screening and management pathways for many non‐HIV age‐related conditions and ultimately missed opportunities to identify declines in health early. To this end, WHO's Model for Healthy Ageing provides an alternative approach to identify vulnerable individuals as they grow older by focusing on their functional ability rather than the absence or presence of clinical disease [5]. The Integrated Care for Older People (ICOPE), the framework which operationalizes this concept, proposes we screen an individuals’ intrinsic capacity (IC) which encompasses all of the physical and mental attributes an individual draws on to function [6]. The construct of IC was developed based on empirical evidence of factors associated with functional loss as an individual ages [7, 8], and since its conception has been validated in multiple studies in the general older population and shown to predict increased care dependence, frailty and mortality [9−12]. Assessments of IC can be performed by non‐specialists, community care providers or through self‐assessments [13, 14], making this care approach attractive for implementation in resource‐limited settings. Despite the wide validation of this construct in the general older population, its clinical utility as a measure to identify PWH with increased vulnerabilities where disability is prevalent [15] has not been studied.

Thus, in this cross‐sectional study, we aimed to optimize the measure of IC in a cohort of adult PWH on ART undergoing routine HIV care in Malaysia and explore its association with the primary health outcomes of frailty and difficulties in performing instrumental activities of daily living (IADL) and secondarily with a range of patient‐reported health outcomes (PROMs). We additionally explored the correlation of IC scores with immune activation markers previously shown to be associated with morbidity and mortality in PWH [16, 17]. Finally, we explored the performance of IC score in an independent cohort of ageing PWH recruited in Hong Kong (HK). This is the first description of the utility of measuring IC among PWH across diverse economic settings and highlights its potential as a tool to integrate functional assessments as part of routine HIV care.

## 2 METHODS

### 2.1 Study population and design

The Malaysian HIV and Ageing cohort is an ongoing, prospective cohort study examining the profile of age‐associated comorbidities and geriatric syndromes in individuals with and without HIV. The first wave of recruitment was conducted between 2014 and 2016 [18], and the current study utilizes cross‐sectional data from the second wave (2021−2023) because not all variables to operationalize the measurement of IC were available in Wave 1. A total of 213 PWH and 108 controls were recruited in Wave 2 (see Figure S1 for participant disposition).

The study was approved by the Ethics Review Board, University Malaya Medical Centre (IRB 2020722–8907), and all participants provided written, informed consent.

### 2.2 Recruitment and study assessments

PWH were recruited during their routine HIV clinic follow‐up at the University Malaya Medical Centre, Malaysia. The inclusion criteria were age 35 years and above, on ART for at least 12 months, and no acute illness at recruitment. The pool of participants (cases and controls) from Wave 1 were approached for recruitment in Wave 2 till target numbers were achieved (Wave 2 recruited fewer participants than Wave 1 due to limited funding). Assessments included biochemical laboratory tests, performance‐based physical assessments and questionnaires (see Table S1 for a list of study assessments). Questionnaires were executed in the participants language of preference either self‐administered electronically, paper‐based or assisted by trained study personnel (if needed), according to standard operating procedures which were periodically reviewed and updated to ensure fidelity. Controls underwent the same set of assessments as PWH in addition to a rapid HIV test (Alere, Japan) prior to recruitment.

### 2.3 Measurement and selection of IC indicators

Domains of IC included vitality, sensory (both vision and hearing), mobility, cognition and mood. Despite consensus on the five domains which make up IC, there is currently a lack of agreement in the literature on the specific indicators to be used to assess deficits in each domain [19, 20]. Thus, we considered the primary indicators measured in the cohort that best fit the conceptual framework of the IC construct and the practical aspects of its execution in our clinic to derive a secondary dataset for this analysis. The selection of indicators was guided by the following considerations: (1) the indicators and thresholds used to define abnormalities have previously been validated in local or regional studies among PWH or older individuals without HIV; (2) the indicators could be assessed with minimal logistical challenges in resource‐limited settings and by non‐specialist providers; and (3) the indicators did not classify a significant majority (>50% of those assessed) as having abnormalities and/or requiring referrals as this would be impractical for implementation. The list of indicators was discussed with a team of clinical and research experts from HIV, primary care, geriatrics and mental healthcare and finalized through consensus. For the vitality domain which reflects the interaction of multiple physiological systems and forms the basis of an individual's biological reserve, we opted to be exhaustive and include all the parameters collected in the study which were listed among the consensus attributes for this domain in a recent WHO guidance document [21]. This included parameters reflecting an individual's level of energy and metabolism, neuromuscular function and immune/stress response, all of which have been shown to be altered in the context of HIV [22−24]. The list of study assessments considered as IC domain indicators and reasons for exclusion (if not selected) are summarized in Table S1.

### 2.4 Measurement of primary outcomes

Frailty and impairment in IADL were chosen as primary outcomes, similar to other studies in the general older population [8, 10, 25, 26]. Frailty was assessed using frailty phenotype as previously described [27], while IADL was estimated using a modified 6‐item assessment of daily functioning from WHODAS 2.0 and SF36 (details in Table S2).

### 2.5 Measurement of other variables and PROMs

Participants’ background characteristics including socio‐demographics and medical history were collected through self‐reports and from reviews of medical charts (for PWH) using standardized data extraction forms by trained personnel. PROMs were assessed using standard validated tools including quality of life, self‐reported health, disability, social participation and loneliness (see Table S2 for details).

### 2.6 Measurement of immune activation markers

Immune activation markers IL‐6, sCD14 and sCD163 were measured in plasma samples isolated from whole blood. IL‐6 and sCD163 were assayed using the ELLA platform (Protein Simple, USA) and sCD14 using sandwich ELISA (R&D Systems, USA) as previously described [28].

### 2.7 Independent cohort of PWH

The protocol and recruitment for Wave 2 of the MHIVA study was part of a larger consortium of cohorts exploring issues of HIV and Ageing in the Asia‐Pacific region. Thus, standard protocols were utilized across study sites and training on assessments was performed to harmonize data collection across sites. For this analysis, we leveraged the availability of similar study parameters from the Prince of Wales Hospital, Hong Kong as an independent cohort of PWH to explore the performance of IC score to predict frailty and loss of IADL. The cohort from HK was chosen given the experience of its investigators in ageing research, the different country income status from Malaysia (high vs. upper‐middle income) and the availability of complete data at the point of analysis. Except for cognitive impairment which utilized thresholds for impairment previously validated in each site [29, 30], we applied the same definitions for impairment for all other indicators to define domain deficits and compute the aggregate IC score.

### 2.8 Statistical analysis

#### 2.8.1 IC score construction

We approached the construction of IC score in this study conceptualizing it as a composite measure where the component domains are treated as formative indicators (causal indicators) [31]. Weighing the practical implementation of this tool in clinic, we opted to use a simple scoring approach that allowed real‐time computation with each domain contributing equal weight to the final composite score as previously described [32]. A score of 1 was assigned to each domain without an abnormality and 0 if there was a deficit. When more than one indicator was included to represent a single domain (specifically for mood and vitality), an average score was computed for that domain (see example in Figure S2). Each domain contributed a maximum score of 1 and the final aggregate IC score ranged from 0 to 6 with higher scores indicating better intrinsic capacities.

#### 2.8.2 Association of IC with primary and secondary PROMs

Logistic regression models adjusted for age, sex, ethnicity and number of chronic comorbidities were used to explore the independent association of aggregate IC scores with the primary outcomes of frailty and IADL impairment in PWH. Additionally, linear regression analysis with three incremental models was performed to assess the association between the IC score with secondary PROMs. Model 1 was unadjusted; Model 2 adjusted for age, sex and ethnicity, and Model 3 adjusted for age, sex, ethnicity, education and employment status.

#### 2.8.3 Association with immune activation markers

Spearman correlations were performed to explore the correlations between IC scores and sCD14, sCD163 and IL‐6. The Kruskal−Wallis test was used to explore if immune activation markers were associated with the number of domain deficits.

#### 2.8.4 Performance of IC score in an independent cohort of PWH

The performance of the composite IC score to identify individuals with frailty and IADL impairment across the two cohorts was evaluated by area under the receiver operating curve (AUC‐ROC). The performance of IC scores to predict these outcomes was also compared to VACS Index 1.0, another composite score derived from clinical parameters and previously associated with frailty and multimorbidity in PWH [33].

All statistical analyses were performed on STATA/SE 16.1 software (Revision 2023) and a *p*‐value of <0.05 was considered statistically significant.

## 3 RESULTS

### 3.1 Characteristics of the study participants

Of the 321 participants recruited, 301 (PWH = 200 and controls = 101) had completed study assessments for all IC domains to compute aggregate scores. The median age in cases and controls were 50 (43−57) and 50 (39−59) years, respectively (Table 1 ). There were more males among PWH (79% vs. 52%, *p*<0.001) but ethnic distribution was comparable. The median duration living with HIV was 13 (10−18) years and 69% had CD4 T‐cell counts > 500 cells/µl.

**Table 1:** Characteristics of participants from the Malaysian HIV and Ageing cohort (*n* = 301)

Characteristics	HIV positive (*n* = 200)	HIV negative (*n* = 101)	*p*‐value^a^
Age			0.938
Below 50 years, *n* (%)	96 (48.0)	48 (47.5)	
50 years and above, *n* (%)	104 (52.0)	53 (52.5)	
Age (years)	50.0 (43.3−57.0)	50.0 (38.5−59.0)	0.567
Gender, *n* (%)			<0.001^*^
Male	158 (79.0)	52 (51.5)	
Female	42 (21.0)	49 (48.5)	
Ethnicity, *n* (%)			0.671
Malay	34 (17.0)	22 (21.8)	
Chinese	142 (71.0)	65 (64.4)	
Indian	22 (11.0)	13 (12.9)	
Others	2 (1.0)	1 (1.0)	
Highest education, *n* (%)			0.075
Primary or lower	24 (12.0)	7 (6.9)	
Secondary	74 (37.0)	29 (28.7)	
Tertiary	102 (51.0)	65 (64.4)	
Employment status, *n* (%)			0.783
Full‐time work	113 (56.8)	61 (60.4)	
Part‐time work	24 (12.1)	13 (12.9)	
Retired	25 (12.6)	13 (12.9)	
Housewife/unemployed	37 (18.6)	14 (13.9)	
Household income, *n* (%)			0.032^*^
≤ RM4850	125 (64.1)	47 (46.5)	
≤ RM4851−RM10,970	52 (26.7)	41 (40.6)	
≤ RM10,971−RM15,040	11 (5.6)	8 (7.9)	
≥ RM15,040	7 (3.6)	5 (5.0)	
Alcohol intake, *n* (%)			0.110
Never	116 (58.0)	50 (49.5)	
Monthly or less	61 (30.5)	43 (42.6)	
2−4 times per month	12 (6.0)	7 (6.9)	
2−3 times per week	5 (2.5)	1 (1.0)	
4+ times per week	6 (3.0)	0 (0.0)	
Smoking status, *n* (%)			<0.001^*^
Never	99 (49.5)	82 (81.2)	
Ex‐smoker	43 (21.5)	13 (12.9)	
Current smoker	58 (29.0)	6 (5.9)	
Low physical activity (IPAQ)^b^, *n* (%)	45 (22.5)	17 (16.8)	0.251
Low haemoglobin (g/l), *n* (%)			
Male <130 g/l	18 (11.4)	1 (1.9)	0.049^*^
Female <120 g/l	17 (40.5)	11 (22.4)	0.063
Glucose (mmol/l)	4.7 (4.1−5.4)	4.5 (4.2−4.9)	0.376
Total cholesterol (mmol/l)	5.0 (4.5−5.5)	5.6 (4.7−6.5)	<0.001^*^
HDL (mmol/l)	1.3 (1.1−1.6)	1.4 (1.1−1.7)	0.031^*^
LDL (mmol/l)	3.0 (2.5−3.5)	3.5 (2.8−4.4)	<0.001^*^
eGFR < 60 ml/minute/m^2^, *n* (%)	12 (6.0)	1 (1.0)	0.067
Total protein (g/l)	78.0 (73.0−82.0)	75.0 (73.0−80.0)	0.003^*^
GGT (U/l)	48.0 (30.0−69.0)	26.0 (18.0−42.0)	<0.001^*^
Albumin (g/l)	44.0 (42.0−47.0)	44.0 (43.0−46.0)	0.970
Hepatitis C, *n* (%)	4 (2.0)	0 (0.0)	0.305
Total CD4+ count (cells/µl)			
Nadir	109 (34−264)	−	−
Current	611 (439−800)	−	−
Current CD4+ count >500 cells/µl, *n* (%)	137 (68.5)	−	−
Current CD4+:CD8+ ratio	0.86 (0.62−1.20)	−	−
Duration living with HIV (years)	13.0 (10.0−18.0)	−	−
Baseline viral load	113,723 (46,592−333,585)	−	−
Viral suppression (<200 copies/ml), *n* (%)	193 (96.5)		
History of ADI, *n* (%)	139 (69.8)	−	−
Duration on ART (years)	11.0 (8.0−14.0)	−	−
Current ART regimen, *n* (%)			−
NNRTI‐based	160 (80.0)	−	
PI‐based	21 (10.5)	−	
INSTI‐based	16 (8.0)	−	
Others	3 (1.5)	−	
History of treatment failure^b^, *n* (%)	16 (8.0)	−	−
Exposure to D‐drugs^b^, *n* (%)	131 (66.2)	−	−

*Note*: Data presented in median (interquartile range, IQR), unless stated otherwise.

Abbreviations: ADI, AIDS‐defining illness; ART, antiretroviral therapy; eGFR, estimated glomerular filtration rate; GGT, gamma‐glutamyl transpeptidase; HDL, high‐density lipoprotein; INSTI, integrase strand transfer inhibitor; LDL, low‐density lipoprotein; NNRTI, non‐nucleoside reverse transcriptase inhibitor; PI, protease inhibitor; RM, Ringgit Malaysia.

^a^ Chi‐square or Fisher exact test performed for categorical variables and Mann−Whitney test for continuous variables.

^b^ Low physical activity is defined as < 600 metabolic equivalent (MET)—minutes per week; History of treatment failure is defined as two consecutive viral load of >1000 copies/ml while on ART; D‐drugs include exposure to didanosine, stavudine, zalcitabine and zidovudine.

* Significant *p*‐value.

**Table 2:** Comparison of indicators used to measure IC domains and health outcomes in PWH (*n* = 200) and controls (*n* = 101) from the Malaysian HIV and Ageing cohort

Characteristics	HIV positive (*n* = 200)	HIV negative (*n* = 101)	*p*‐value^a^
IC composite score	5.47 (4.80−5.80)	5.60 (4.80−5.80)	0.093
COGNITIVE DOMAIN			
Impaired MOCA (demographic‐adjusted), *n* (%)	50 (25.0)	13 (12.9)	0.015^*^
SENSORY DOMAIN (VISION AND HEARING)			
Self‐reported vision impairment (VF14), *n* (%)	7 (3.5)	2 (2.0)	1.000
VF14 score	92.9 (87.7−97.9)	93.8 (80.9−97.8)	0.897
Self‐reported hearing impairment (HHIE), *n* (%)	2 (1.0)	1 (1.0)	1.000
HHIE score	5.0 (2.0−25.0)	4.0 (3.0−19.0)	0.853
MOOD DOMAIN			
DASS‐21, *n* (%)			
Depression (score ≥ 7)	32 (16.0)	10 (9.9)	0.149
Anxiety (score ≥ 6)	37 (18.5)	16 (15.8)	0.567
Stress (score ≥ 10)	13 (6.5)	6 (5.9)	0.851
MOBILITY DOMAIN			
Slow gait speed <0.8 m/second, *n* (%)	19 (9.5)	13 (12.9)	0.370
Gait speed (m/second)	1.1 (1.0−1.2)	1.0 (0.9−1.2)	0.018^*^
VITALITY DOMAIN			
Nutrition status, *n* (%)			0.282
Normal	153 (76.5)	84 (83.2)	
At risk of malnutrition	43 (21.5)	17 (16.8)	
Malnourished	4 (2.0)	0 (0.0)	
HbA1c (%)	5.4 (5.1−5.9)	5.5 (5.3−5.9)	0.139
Abnormal HbA1c >6.5%, *n* (%)	24 (12.0)	8 (7.9)	0.278
Low grip strength, *n* (%)			0.139
Men (<26 kg), women (<18 kg)	64 (32.0)	28 (27.7)	0.447
High hsCRP ≥ 6 mg/l, *n* (%)	24 (12.0)	10 (9.9)	0.587
BMI (kg/m^2^), *n* (%)			0.092
<18.50 (Underweight)	20 (10.0)	5 (5.0)	
18.50−24.99 (Normal)	65 (32.5)	29 (28.7)	
25−29.99 (Overweight)	40 (20.0)	15 (14.9)	
≥30 (Obese)	75 (37.5)	52 (51.5)	
PRIMARY AND SECONDARY HEALTH OUTCOMES			
Frailty phenotype, *n* (%)			0.220
Non frail	67 (33.5)	38 (37.6)	
Prefrail	117 (58.5)	60 (59.4)	
Frail	16 (8.0)	3 (3.0)	
Impaired IADL, *n* (%)	55 (27.8)	18 (18.0)	0.064
VACS index 1.0	12.0 (6.0−22.0)	12.0 (0.0−22.0)	0.598
Number of chronic comorbidities	3 (2−4)	2 (1−3)	0.009^*^
Polypharmacy ≥5 medications,^b^ *n* (%)	74 (37.0)	7 (6.9)	<0.001^*^
WHODAS scores			
Communication and understanding	4.0 (0.0−21.0)	8.0 (0.0−17.0)	0.865
Getting around	0.0 (0.0−10.0)	0.0 (0.0−10.0)	0.304
Self‐care	0.0 (0.0−0.0)	0.0 (0.0−0.0)	0.318
Getting along with people	5.0 (0.0−20.0)	5.0 (0.0−15.0)	0.404
Life activities	3.0 (0.0−19.0)	0.0 (0.0−11.0)	0.063
Participation in society	13.0 (3.0−28.0)	3.0 (0.0−13.0)	<0.001^*^
WHOQOL‐HIV‐BREF score			
Physical	69.0 (56.0−81.0)	75.0 (66.0−86.0)	0.031^*^
Psychological	65.0 (59.0−76.0)	75.0 (65.0−79.0)	0.003^*^
Independence	75.0 (56.0−81.0)	−	−
Social	63.0 (50.0−75.0)	75.0 (58.0−75.0)	0.001^*^
Environment	66.0 (56.0−75.0)	72.0 (63.0−78.0)	0.022^*^
Spirituality	69.0 (50.0−88.0)	−	−
CASP‐19 score			
Control	11.0 (10.0−13.0)	12.0 (11.0−14.0)	0.001^*^
Autonomy	14.0 (12.0−16.0)	15.0 (14.0−18.0)	0.001^*^
Pleasure	18.0 (15.0−20.0)	19.0 (17.0−20.0)	0.001^*^
Self‐realization	16.0 (14.0−19.0)	18.0 (15.0−20.0)	0.004^*^
Self‐rated health score	2.0 (2.0−3.0)	2.0 (2.0−3.0)	0.751
Lubben Social Network Scale			
Family isolation	7.0 (4.0−9.0)	9.0 (6.0−11.0)	<0.001^*^
Friendship isolation	7.0 (5.0−9.0)	9.0 (6.0−11.0)	0.001^*^
High risk of social isolation, *n* (%)	90 (45.0)	22 (22.0)	<0.001^*^
De Jong Loneliness Scale			
Emotional loneliness	1.0 (0.0−2.0)	1.0 (0.0−1.0)	<0.001^*^
Social loneliness	2.0 (1.0−3.0)	2.0 (0.0−3.0)	0.032^*^
Overall loneliness	3.0 (2.0−5.0)	2.0 (1.0−4.0)	<0.001^*^
Lonely status, *n* (%)	157 (78.9)	66 (65.3)	0.011^*^

*Note*: Data presented in median (interquartile range, IQR), unless stated otherwise.

Abbreviations: BMI, body mass index; CASP‐19, Control, Autonomy, Self‐Realization and Pleasure quality of life scale; DASS‐21, Depression Anxiety and Stress Scale 21; HHIE, Hearing Handicap Inventory for the Elderly; hsCRP, high‐sensitivity C‐reactive protein; IADL, instrumental activities of daily living; IC, intrinsic capacity; MOCA, Montreal Cognitive Assessment; PWH, people living with HIV; VACS, Veterans Aging Cohort Study; VF‐14, Visual Function Index; WHODAS, WHO Disability Assessment Schedule; WHOQOL‐HIV‐BREF, WHO Quality of Life‐HIV BREF.

^a^ Chi‐square or Fisher exact test performed for categorical variables and Mann−Whitney test for continuous variables.

^b^ 5 or more medications excluding antiretrovirals.

* Significant *p*‐value.

**Figure 1:**
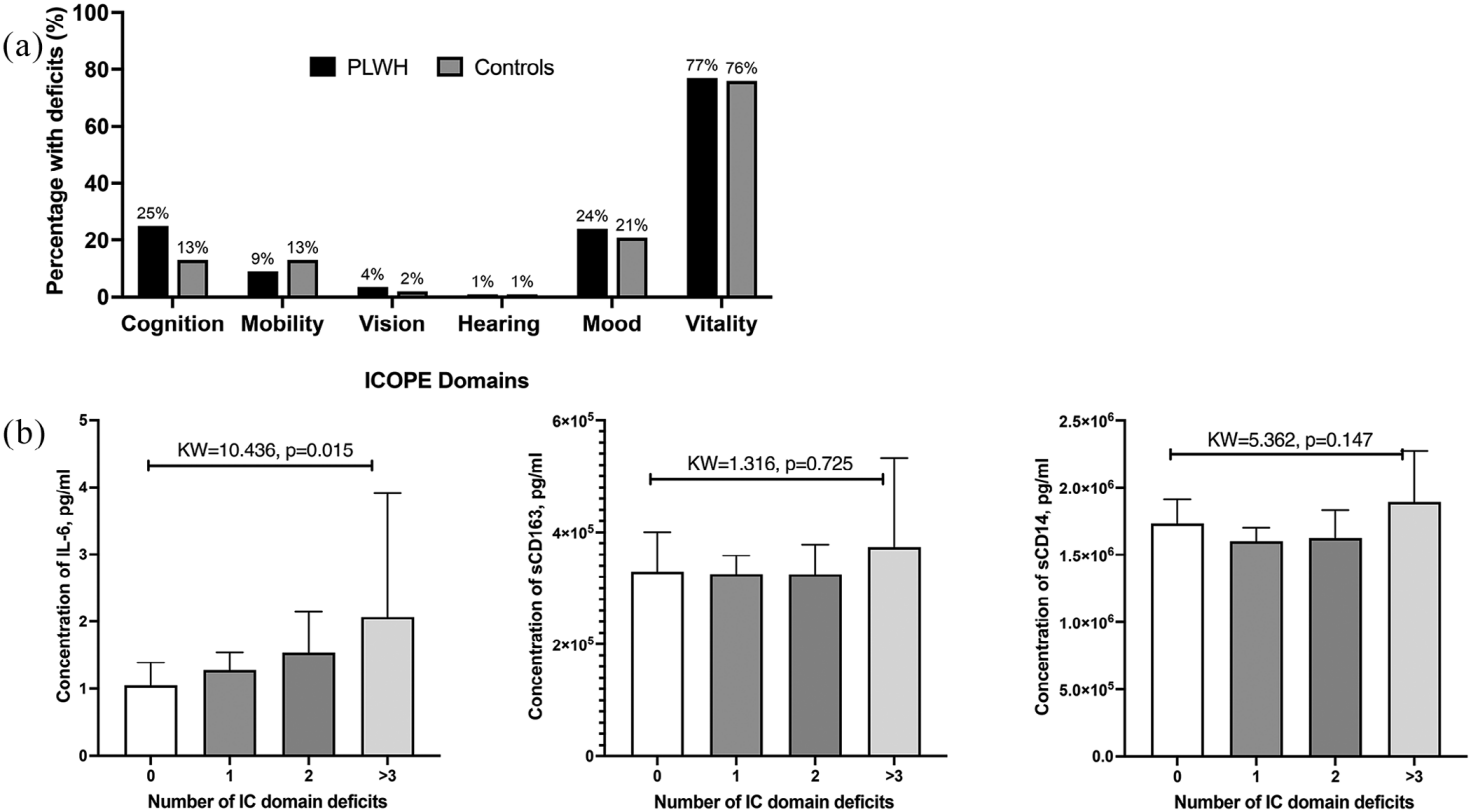
Frequency of deficits in intrinsic capacity domains in PWH and controls, and its association with markers of immune activation in PWH. (a) Bar graph demonstrating difference in frequencies of PWH (*n* = 200) and controls (*n* = 101) experiencing deficits in intrinsic capacity (IC) domains and (b) comparison of immune activation markers across the number of IC deficits in PWH. Comparisons were performed using Kruskal−Wallis (KW) test for group comparisons. A *p*‐value of <0.05 was considered significant and error bars correspond to median and interquartile range.

When IC domains were compared (Figure 1a), the proportion reporting deficits was numerically higher in PWH compared to controls and statistically significant for cognitive test performance (25% vs. 13%, *p*<0.015). PWH had lower median IC aggregate scores compared to controls but the difference was not statistically significant (5.4 vs. 5.6, *p* = 0.093) (Table 2).

### 3.2 Association of composite IC score with frailty, IADL and other patient‐reported outcomes in PWH

Logistic regression analysis adjusted for age, sex, ethnicity and number of chronic comorbidities found increased aggregate IC scores associated with the reduced odds of frailty (OR 0.17 95% CI 0.07−0.42, *p*<0.001) and IADL impairment (OR 0.25 95% CI 0.14−0.46, *p*<0.001) in PWH. When analysis of associations between aggregate IC scores and PROMs was performed (Table 3), IC scores were significantly associated with all the outcomes assessed with the *R* ^2^ displaying a general trend of increase in adjusted models (Model 1: unadjusted; Model 2: adjusted for age, sex and ethnicity; Model 3: adjusted for age, sex, ethnicity, education and employment) and ranging between 7% and 23% across the different outcomes in the fully adjusted model.

**Table 3:** Linear regression analysis of composite IC scores with patient‐reported health outcomes in PWH (*n* = 200)

Health outcome (tool)	Model 1				Model 2				Model 3			
	B	95% CI	*R* ^2^	*p*‐value	B	95% CI	*R* ^2^	*p*‐value	B	95% CI	*R* ^2^	*p*‐value
Functional ability (WHODAS)	−8.32	(−10.84, −5.81)	0.18	<0.001	−8.99	(−11.73, −6.26)	0.20	<0.001	−10.31	(−13.15, −7.48)	0.23	<0.001
Number of chronic comorbidities	−0.98	(−1.31, −0.65)	0.14	<0.001	−0.80	(−1.15, −0.45)	0.18	<0.001	−0.77	(−1.14, −0.40)	0.18	<0.001
Number of medications^a^	−0.97	(−1.41, −0.54)	0.08	<0.001	−0.73	(−1.19, −0.27)	0.15	0.002	−0.74	(−1.21, −0.26)	0.18	0.002
Quality of life for older people (CASP‐19)	3.37	(1.59, 5.15)	0.06	<0.001	3.49	(1.58, 5.39)	0.10	<0.001	3.52	(1.53, 5.51)	0.10	0.001
Quality of life for PWH (WHOQOL HIV BREF)	6.76	(3.25, 10.28)	0.07	<0.001	7.65	(3.92, 11.39)	0.14	<0.001	7.65	(3.73, 11.56)	0.15	<0.001
Self‐rated health (WHS)	−0.34	(−0.49, −0.18)	0.08	<0.001	−0.39	(−0.56, −0.22)	0.09	<0.001	−0.40	(−0.57, −0.22)	0.09	<0.001
Loneliness (De Jong Gierveld Loneliness Scale)	−0.55	(−0.91, −0.19)	0.04	0.003	−0.61	(−1.00, −0.22)	0.07	0.002	−0.52	(−0.92, −0.11)	0.07	0.013
Social participation (Lubben Social Network Scale)	1.47	(0.38, 2.55)	0.03	0.008	1.99	(0.83, 3.15)	0.08	0.001	1.27	(0.15, 2.40)	0.20	0.027

Model 1 = unadjusted model.

Model 2 = adjusted for age, gender and ethnicity.

Model 3 = adjusted for age, gender, ethnicity, education and employment.

*Note*: Multiple linear regression was applied. There was no interaction and multicollinearity problem. All the assumptions (normality, linearity and homoscedasticity) were met for all models.

Abbreviations: B, beta coefficient; CASP‐19, Control, Autonomy, Self‐Realization and Pleasure quality of life scale; CI, confident interval; PWH, people living with HIV; WHODAS, WHO Disability Assessment Schedule; WHOQOL‐HIV‐BREF, WHO Quality of Life‐HIV BREF.

^a^ Medications include antiretroviral and non‐antiretroviral medications.

### 3.3 Correlation of composite IC scores and immune activation markers

IC scores correlated significantly with IL‐6 levels (*r* = −0.225, *p* = 0.002), demonstrated a trend with sCD14 (*r* = −0.127, *p* = 0.075) but not with sCD163 (*r* = −0.107, *p* = 0.137). Additionally, IL‐6 levels increased with a higher number of domain deficits (KW = 10.436, *p* = 0.015) but this was not observed with sCD14 (KW = 5.362, *p* = 0.147) and sCD163 (KW = 1.316, *p* = 0.725) levels (Figure 1b).

### 3.4 Performance of aggregate IC score in predicting frailty and IADL in an independent cohort of PWH

We next applied the composite IC scoring in an independent cohort of PWH recruited in HK using the same study protocol. We evaluated the predictive capacity of the IC score in both cohorts using the AUC‐ROC. Compared to Malaysia, participants from HK were older, less educated, had higher income levels, acquired HIV more recently and were less immunocompromised at ART initiation (Table S3). In terms of IC domains, fewer participants from HK had cognitive impairment and symptoms of anxiety compared to participants from Malaysia (Figure S3). Median IC aggregate scores were comparable in both cohorts (5.6 vs. 5.5, *p* = 0.220), as were the primary health outcomes of frailty and IADL impairment (Table S4).

**Figure 2:**
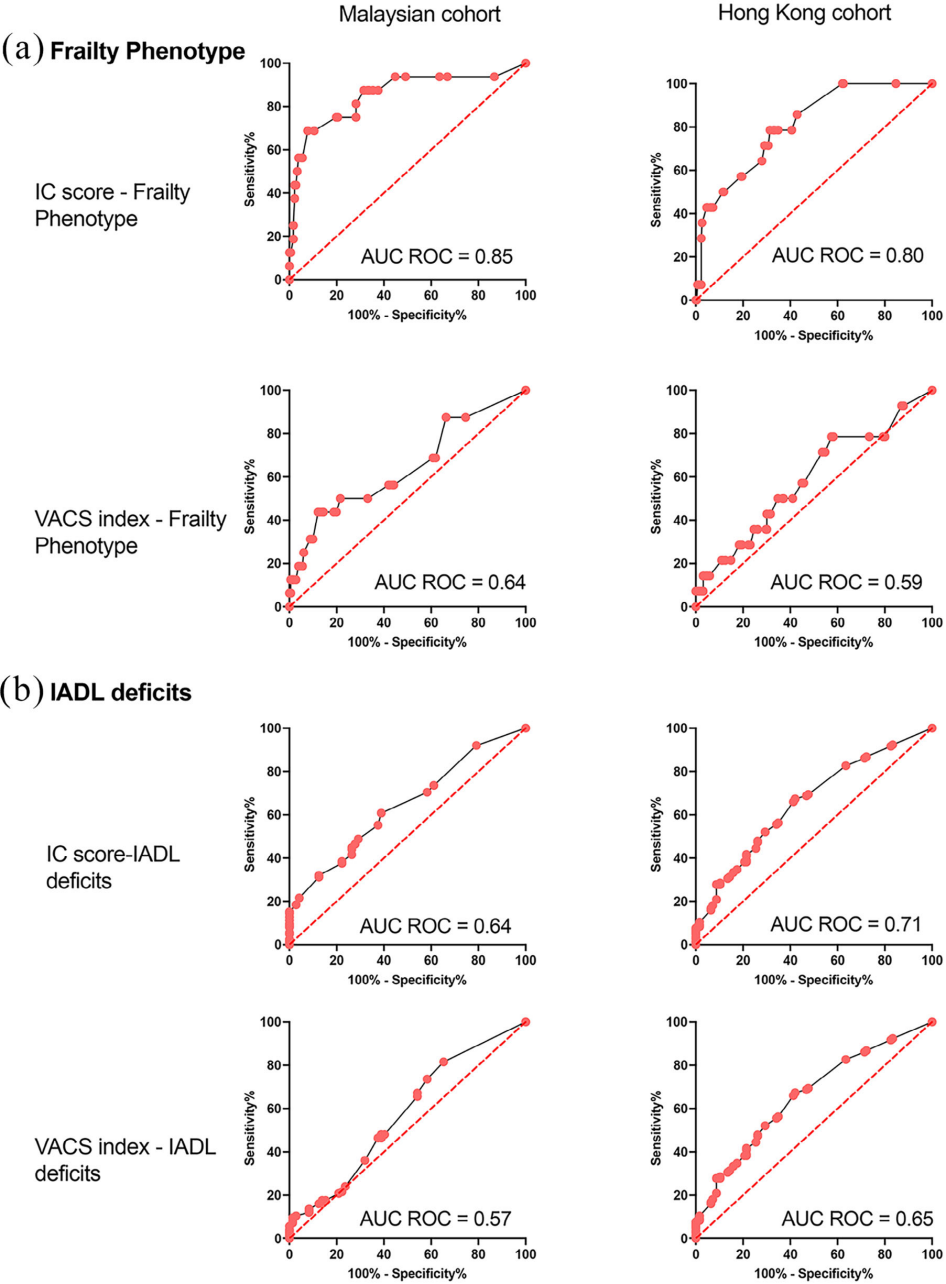
Performance of aggregate intrinsic capacity (IC) scores and VACS index 1.0 in identifying PWH with frailty and deficits in instrumental activities of daily living (IADL deficits) in two independent cohorts from Malaysia and Hong Kong. Area under the ROC curve for intrinsic capacity (IC) scores and VACS 1.0 with the outcomes of (a) frailty and (b) instrumental activities of daily living (IADL) in the Malaysian (left panel) and Hong Kong (right panel) cohorts.

In analysis assessing the performance of IC scores, we found aggregate IC scores predicted frailty well in both the Malaysian (AUC‐ROC: 0.85; 95% CI = 0.73−0.97) and HK cohorts (AUC‐ROC: 0.80; 95% CI = 0.71−0.91) and better than VACS 1.0 index (Malaysian AUC‐ROC: 0.64; 95% CI = 0.48−0.80 and HK AUC‐ROC: 0.59; 95% CI = 0.43−0.75) (Figure 2a). Similarly, IC scores predicted impairment in IADL better than VACS 1.0 index in both cohorts, albeit with a lower discriminatory ability than for frailty (Figure 2b).

## 4 DISCUSSION

This is the first study, to our knowledge, to evaluate the utility of IC to identify PWH with physical vulnerabilities in two independent cohorts. The ability of IC scores to discriminate individuals with frailty well and those with IADL deficits more moderately across two economically diverse settings is consistent with findings in the general older population (>60−65 years) [20], and extends this evidence to middle‐aged PWH. Its correlation with IL‐6, an established marker of inflammaging, further confirms its biological relevance in PWH. The independent association of compromised IC, denoted by lower composite scores, with multiple adverse health outcomes spanning clinical, physical and social domains also demonstrates the potential utility of this functional construct to identify PWH with increased health needs independent of their HIV‐related outcomes. The composite IC scores derived in this study utilized assessments that can be performed by non‐specialist healthcare workers in a contemporary HIV clinic setting making this tool extremely attractive for implementation in settings where the ability to perform comprehensive geriatric assessments as part of routine HIV care is limited.

When comparing individual domains in the Malaysian cohort, we found significantly higher frequencies of PWH demonstrating deficits in the cognitive domain compared to controls, consistent with prior studies in our setting [29, 34]. No differences were found in the other domains, noting that cases and controls were not entirely matched for sex, household income and lifestyle factors in the final analysis. This was not entirely surprising given the relatively young age of the participants and the context of recruitment in an ambulatory care setting. However, despite minimal differences in individual domain deficits, we found a consistent significant association between composite ICOPE scores with frailty and IADL in two independent cohorts which somewhat supports the robustness of this construct even in a population where there is significant heterogeneity in how HIV‐specific factors may influence the biological process of ageing throughout their life course including the age of HIV acquisition [35] and duration of viraemia [36]. The assessment of IC was developed with the intention to monitor trajectories of ageing across the second half of life and as a means to identify vulnerable individuals early, prior to the occurrence of frailty and increased care dependence [37]. In this regard, our findings from this cross‐sectional analysis provide strong justification for future validation studies in longitudinal cohorts of PWH from diverse settings to establish the capacity of IC to predict poor health outcomes including age thresholds where screening may be appropriate and cost‐effective.

There have been a number of clinical composite ageing markers previously tested in the context of HIV including VACS index and PhenoAge, and shown to be associated with or predict all‐cause mortality, frailty and age‐related comorbidities [38, 39]. These assessments utilize clinical/biochemical parameters routinely measured in clinical care and related to organ injury, reflecting disease‐based outcomes of ageing. Although we found IC scores performed better in identifying frail PWH compared to VACS index 1.0 in both the Malaysian and HK cohorts, these composite scores likely measure different aspects of ageing and their role in HIV clinical care may differ. However, from a person‐centred care perspective [40], monitoring and enquiring about functional changes during clinical consultations is likely to be more meaningful and relatable to an ageing person compared to discussions around changes in laboratory values. Additionally, assessments of IC also provide a starting point for defined care pathways in those demonstrating deficits in specific domains (see WHO ICOPE manual https://www.who.int/publications/i/item/WHO‐FWC‐ALC‐19.1), and this may be attractive for HIV care programmes which otherwise lack the capacity and know‐how to plan for integrated geriatric services as part of routine HIV care. Thus, assessments of IC may be better suited for HIV programmes wanting to pivot to integrated care services beyond disease‐based monitoring and helps align programmes to be more person‐centred.

Few studies to date have explored how blood‐based biomarkers track with aggregate IC scores (recently reviewed in [41]). It is well established that PWH experience increased immune activation despite long‐term ART and especially in those with a history of advanced HIV disease [42]. Markers including IL6, sCD14, sCD163, sTNFR1, D‐dimer and kynurenine to tryptophan (KT) ratio have all been associated with increased age‐related morbidity and mortality [16, 43]. In our analysis, we found IL‐6 was significantly correlated with lower composite IC scores and individuals with a higher number of domain deficits had higher IL6. Studies in the general older population (>60 years) have consistently shown IL‐6 to be associated with reduced IC scores [41] as well as faster declines in IC trajectories [44]. IL‐6 is a classical marker of chronic inflammation and is associated with multiple age‐related comorbidities and geriatric syndromes in both people with and without HIV [45]. Its association with the aggregate IC scores in this relatively young cohort of PWH affirms IC as a comprehensive measure of functional ageing in this population. sCD14, a marker of monocyte activation, and sCD163, a marker of macrophage activation, have previously been associated with mortality and increased cardiovascular events in PWH [17] but have not been studied in the context of IC in the general population. We speculate that its lack of association with aggregate IC scores may reflect the role of myeloid cell activation in HIV‐specific pathologies accentuating age‐related comorbidities rather than ageing in general but this should be confirmed in larger cohort studies.

The strengths of this study are the assessment of the IC construct with a variety of health outcomes and the confirmation of its utility in two independent clinic cohorts of PWH. However, there are some important limitations too. First, this was the secondary analysis of a comprehensive dataset where parameters aligned with the domains of IC were selected to compute IC scores. How alternative parameters, if available, would have influenced the performance of the tool is not entirely clear. However, there is currently no consensus on which parameters to use to define domain deficits or to compute IC composite scores [46] and a wide variety of parameters and approaches have been used in published studies. The WHO ICOPE manual provides a recommendation of assessments but highlights this should be tailored to individual site capacity. Future validation studies should ideally test practical and easy‐to‐implement assessments, most‐suited for their settings. Secondly, the WHO ICOPE framework is a two‐step process that utilizes an initial brief set of screening questions (2−3 questions per domain) before performing a complete assessment of IC in those demonstrating some degree of deficit [47]. We did not perform this initial screening and applied a full assessment in all participants given our aim to assess the performance of IC measures in PWH. For implementation purposes, more research on the sensitivity and specificity of these brief screening questions is needed especially when applied in a younger cohort of PWH whose functional deficits may be more subtle. Implementation studies exploring the training needs for non‐specialist staff, and the feasibility and acceptability of integrating ICOPE into HIV clinic flow are also needed. Thirdly, some parameters used to assess IC were closely linked/overlapped with frailty phenotype measures and could potentially have influenced our findings. To confirm this, we modified our calculations of IC by omitting grip strength from the vitality assessment and replacing gait speed with five times chair stand. In this modified analysis, IC scores (IC) remained significantly correlated with outcomes of frailty (OR 0.23 95% CI 0.10−0.52, *p*<0.001) and IADL impairment (OR 0.38 95% CI 0.23−0.62, *p*<0.001) after adjustments for age, sex, ethnicity and number of chronic comorbidities. Finally, this was a cross‐sectional analysis in a limited number of participants and thus the utility of monitoring IC trajectories to predict adverse outcomes in ageing PWH cannot yet be established.

## 5 CONCLUSIONS

We found the measurement of IC derived from the assessments of five functional domains sufficient to identify PWH with poor physical and social outcomes, consistent with WHO's conceptual model for Healthy Ageing. The parameters used to assess these domains are easy to perform by trained non‐specialist staff, making this measurement attractive for integration into HIV care in resource‐limited settings where the majority of PWH reside. More research, however, is needed in longitudinal cohorts to assess the predictive capacity of monitoring IC over the longer‐term (5‐ and 10‐year horizons) as well as its sensitivity to interventions in PWH.

## COMPETING INTERESTS

The authors have no competing interests to declare.

## AUTHORS’ CONTRIBUTIONS

RR, GC‐YL, SBK, PLW and MN conceived the study; SH, JSL, JYC, WYH, MLC, SFSO, CC and VW contributed to recruitment and data collection; RR, PLW, SBK, KH, RCY, MN, SH, CC, VW and GC‐YL analysed the data; YSH and NSZ performed all the laboratory analysis; RR and SYH wrote and formatted the manuscript; RR, GC‐YL and PLW contributed to the funding. All authors reviewed and approved the final manuscript.

## FUNDING

This work was supported by an educational grant from Gilead Sciences and GSK. The funders have had no role in the design and execution of the study, analyses, data interpretation or decision to submit.

## Supporting information



Supporting Information

## Data Availability

The data that support the findings of this study are available from the corresponding author upon reasonable request.
