# Supplementary material for: Assessing intrinsic capacity for person‐centred HIV care: a cross‐sectional study in ageing populations in Malaysia and Hong Kong
Source: J Int AIDS Soc. 2024 Dec 26;28(1):e26404. doi: 10.1002/jia2.26404 (PMC11670474; doi:10.1002/jia2.26404)
Supplement: Supplementary file 1 — Supporting Information [file JIA2-28-e26404-s001.docx]

**Supplementary Materials**

Supplementary tables

Supplementary table 1: Table of all study assessments, definitions of abnormality and selection as preferred indicators to compute IC composite score following literature review and group consensus.

| **Domains** | **Indicators** | **Definition for abnormality** | **Selected for IC composite score?**  **Yes(Y)/No(N)** | **Reason for exclusion** |
| --- | --- | --- | --- | --- |
| Cognition | MOCA (standard cutoff) | Raw score < 26 | N | Not validated in the local population |
| Cognition | MOCA (demographically- adjusted) | Global deficit score ≥ 0.5 | Y |  |
| Mood | PHQ9 | Raw score 10-27 | N | Only captured individuals with depression while other mood disorders like anxiety and stress are also highly prevalent among PWH. |
| Mood | DASS21 | Raw score: Stress 10-21, Depression 7-21, Anxiety 6-21 | Y |  |
| Hearing | Self-reported hearing problem and HHIE | Score > 8 following a self-report of hearing problems | Y |  |
| Vision | Visual questionnaire | study participant self-reported having not good/poor vision | Y |  |
| Vision | Self-reported poor vision and VF-14 | Degree of impairment ≥ 3 (moderate to worse impairment) | Y |  |
| Vision | Logmar | Moderate to worse impairment in at least one eye measured using Snellen Chart (reading converted to LogMar) | N | Assessment in clinic requires additional space. |
| Mobility | SPPB | score 0-8 | N | Assessment in clinic requires additional space and takes time to perform. |
| Mobility | Gait speed (AWGS 2014) | Speed < 0.8m/s | Y |  |
| Mobility | Gait Speed (AWGS 2019) | Speed < 1.0m/s | N | Thresholds identified over 50% of participants as having abnormal gait speed |
| Vitality | Mini Nutritional Assessment | Score 0-11 | Y |  |
| Vitality | BMI (Obese/underweight) | Underweight < 18.5, Obese ≥ 25 | Y |  |
| Vitality | Grip strength (AWGS 2014) | Male < 26kg, Female < 18kg | Y |  |
| Vitality | Grip Strength (AWGS 2019) | Male < 28kg, Female < 19kg | N | Thresholds identified over 50% of participants as having abnormal grip strength |
| Vitality | hsCRP | hsCRP ≥ 6mg/L | Y |  |
| Vitality | Appendicular skeletal muscle mass^#^ | Low muscle mass <7.0kg/m^2^ for men; <5.7kg/m^2^ women | N | Requires BIA machine and incurs additional cost to service and purchase electrodes |
| Vitality | Skeletal muscle mass index^##^ | Ratio ≥0.75 | N | Requires BIA machine and incurs additional cost to service and purchase electrodes |
| Vitality | HbA1C | HbA1C reading > 6.5% | Y |  |
| Vitality | Unintentional weight loss i.e weight loss of > 5 pounds in the past 12 months | Questionnaire-based | N | Overlapped with the definition of frailty measured by frailty phenotype |

^#^Appendicular skeletal muscle mass (ASM) was calculated as follows: Male ASM = [0.197 x (impedance index) + 0.179 x (weight) – 0.019]; female ASM = 0.221 x (impedance index) + 0.117 x (weight) + 0.881. Impedance index was estimated the ratio of height squared divided by the resistance (height^2^/R).

^##^Skeletal muscle mass index was calculated as ASM/height^2^ (kg/m^2^).

Abbreviation: AWGS, Asian Working Group for Sarcopenia; BIA, bioelectrical impedance analysis; BMI, body mass index; DASS-21, Depression Anxiety and Stress Scale 21; HHIE, Hearing Handicap Inventory for the Elderly; hsCRP, high-sensitivity C-reactive protein; IC, intrinsic capacity; MOCA, Montreal Cognitive Assessment; PHQ-9, Patient Health Questionnaire-9; PWH, people living with HIV; SPPB, Short Physical Performance Battery; VF-14, Visual Function Index.

Supplementary table 2: Tools used to assess primary and secondary health outcomes and definition for abnormalities.

| **Assessment** | **Tool description and scoring** | **Reference** |
| --- | --- | --- |
| Frailty phenotype | Sum of scores based on grip strength, gait speed, loss of more than 10 lbs in last 12 months, calories burnt per week and exhaustion questions. Any abnormalities was scored as 1. Total scores were categorised as follows; 0 is non frail, 1-2 is prefrail, 3 and above is frail. | Fried et al. (2001) |
| Instrumental activities of daily living (IADL) | Modified version of IADL derived from participants’ responses to WHODAS 2.0 and SF36 questionnaires. Assessments included difficulties to get to places out of walking distance, shopping for groceries or clothes, doing house work by your own, doing heavy work around the house, walk up and down the stairs and walking a mile without help. Any abnormalities was scored as 1. Analysis was done based on presence of moderate to severe impairment (score 1) and no or mild impairment (score 0). | Lawton & Brody (1969) |
| Functional ability | WHO Disability Assessment Schedule 2.0 (WHODAS 2.0) is a generic assessment instruments that measures health and disability of participants. The questionnaire comprises six domains of life with a five-point Likert-type scale. Domain 1 assesses understanding and communication, specifically concentration, problem-solving, learning, and communicating. Domain 2 assesses moving and getting around. Domain 3 assesses the ability of self-care including questions on bathing, dressing, eating, and staying alone. Domain 4 assesses getting along with other people and difficulties that might be encountered with this due to a health condition. Domain 5 assesses life activities including difficulties in day-to-day activities, such as household, work, and school activities. Domain 6 assesses participation including how other people and the world around them make it difficult for them to take part in society. The scores for each item were summed within each domain and the summary score was converted into a metric ranging from 0 to 100 (where 0 = no disability, 100 = full disability). | Üstün et al. (2010) |
| Quality of life | The Control, Autonomy, Self-realization, Pleasure (CASP-19) is a 19-item self-report questionnaire used to measure the quality of life in individuals, with a four-point Likert response scales “Often” to “Never”. The score for each domain were summed and higher scores equal better quality of life.  The World Health Organization’s Quality of Life Instrument in HIV infection (WHOQOL HIV-BREF) consists of a total of 31 questions, including two general questions and 29 specific questions explaining six areas of QoL (physical wellbeing, psychological health, independence, social relations, environmental health and spiritual wellness). Domain scores are calculated by taking the mean of all items included in each domain and multiplying by a factor of four. These scores are then transformed to a 0-100 scale. Higher scores equal better quality of life. | Hyde et al. (2003)  O’Connell and Skevington. (2012) |
| Self-rated health | The first question of World Health Survey (WHS), “In general, how would you rate your health today?” was used to examine participants’ general health. The responses were in a five-point Likert response scales “very good = 0” to “very bad = 5”. Lower score equals better health. | Üstün et al. (2003) |
| Social participation | Lubben Social Network Scale (LSNS)-6 includes a set of three items for family and a comparable set of three items for friends, asking about frequency of contact and emotional closeness. The participant answered each question by selecting a value on a 6-point scale (ranging from 0 = none to 5 = nine or more). Higher scores indicate stronger ties with family or friends. A score of 12 (from both friends and family) and lower delineates “at-risk” for social isolation. | Lubben et al. (2006) |
| Loneliness | The De Jong Gierveld Loneliness Scale is based on two-dimensional structure of loneliness comprising social and emotional loneliness. The items are scored on a scale from 0 to 2 and then recoded as dichotomous (0 or 1). For each social and emotional loneliness, the score ranges from 0 to 3, with two categories: not emotionally/socially lonely (score:0) and emotionally/socially lonely (score 1-3). The final score ranges from 0 (no loneliness) to 6 (extreme loneliness), with two categories: no loneliness (scores: 0-1) and loneliness (score equal or higher than 2). | De Jong Gierveld & Van Tilburg, (2006) |

**Reference**

1. Fried LP, Tangen CM, Walston J, Newman AB, Hirsch C, Gottdiener J, Seeman T, Tracy R, Kop WJ, Burke G, McBurnie MA; Cardiovascular Health Study Collaborative Research Group. Frailty in older adults: evidence for a phenotype. J Gerontol A Biol Sci Med Sci. 2001 Mar;56(3):M146-56.
2. Gierveld, J. D. J., & Tilburg, T. V. (2006). A 6-Item Scale for Overall, Emotional, and Social Loneliness: Confirmatory Tests on Survey Data. Research on Aging, 28(5), 582-598.
3. Hyde M, Wiggins RD, Higgs P, Blane DB. A measure of quality of life in early old age: the theory, development and properties of a needs satisfaction model (CASP-19). Aging Ment Health. 2003 May;7(3):186-94.
4. Lawton MP, Brody EM. Assessment of older people: self-maintaining and instrumental activities of daily living. Gerontologist. 1969 Autumn;9(3):179-86.
5. Lubben J, Blozik E, Gillmann G, Iliffe S, von Renteln Kruse W, Beck JC, Stuck AE. Performance of an abbreviated version of the Lubben Social Network Scale among three European community-dwelling older adult populations. Gerontologist. 2006 Aug;46(4):503-13.
6. O'Connell KA, Skevington SM. An international quality of life instrument to assess wellbeing in adults who are HIV-positive: a short form of the WHOQOL-HIV (31 items). AIDS Behav. 2012 Feb;16(2):452-60.
7. Üstün, T. B., Chatterji, S., Mechbal, A., Murray, C. J., & WHS Collaborating Group. (2003). The world health surveys. *Health systems performance assessment: debates, methods and empiricism. Geneva, World Health Organization*, *797*.
8. Ustün TB, Chatterji S, Kostanjsek N, Rehm J, Kennedy C, Epping-Jordan J, Saxena S, von Korff M, Pull C; WHO/NIH Joint Project. Developing the World Health Organization Disability Assessment Schedule 2.0. Bull World Health Organ. 2010 Nov 1;88(11):815-23.

Supplementary table 3: Comparison of participant characteristics from the Malaysian and Hong Kong cohorts of PWH.

| **Characteristics** | **HIV+ Malaysia (n=200)** | **HIV+ Hong Kong (n=275)** | ***p*-value**^@^ |
| --- | --- | --- | --- |
| SOCIAL-DEMOGRAPHICS AND LIFETSYLE | | | |
| Age |  |  | <0.001* |
| Below 50 years, n (%) | 96 (48.0) | 87 (31.6) |  |
| 50 years and above, n (%) | 104 (52.0) | 188 (68.4) |  |
| Age (years) | 50.0 (43.3-57.0) | 56.0 (46.0-64.0) | <0.001* |
| Gender, n (%) |  |  | 0.005* |
| Male | 158 (79.0) | 243 (88.4) |  |
| Female | 42 (21.0) | 32 (11.6) |  |
| Ethnicity, n (%) |  |  |  |
| Malay | 34 (17.0) | - |  |
| Chinese | 142 (71.0) | 270 (98.2) |  |
| Indian | 22 (11.0) | - |  |
| Others | 2 (1.0) | 5 (1.8) |  |
| Education, n (%) |  |  | 0.003* |
| Primary or lower | 24 (12.0) | 49 (17.8) |  |
| Secondary | 74 (37.0) | 128 (46.5) |  |
| Tertiary | 102 (51.0) | 98 (35.6) |  |
| Employment status, n (%) |  |  | 0.020* |
| Full-time work | 113 (56.8) | 134 (48.7) |  |
| Part-time work | 24 (12.1) | 31 (11.3) |  |
| Retired | 25 (12.6) | 66 (24.0) |  |
| Housewife/Unemployed | 37 (18.6) | 44 (16.0) |  |
| Income^#^, n (%) |  |  |  |
| *Malaysia* |  |  |  |
| ≤ RM4,850 | 125 (64.1) | - |  |
| ≤ RM4,851-RM10,970 | 52 (26.7) | - |  |
| ≤ RM10,971-RM15,040 | 11 (5.6) | - |  |
| ≥ RM15,040 | 7 (3.6) | - |  |
| *Hong Kong* |  |  |  |
| < HK$10,000 | - | 113 (41.9) |  |
| HK$10,000- HK$20,000 | - | 82 (30.4) |  |
| HK$20,001- HK$50,000 | - | 58 (21.5) |  |
| > HK$50,000 | - | 17 (6.3) |  |
| *Income classification by individual country census:* |  |  | <0.001* |
| Low | 125 (64.1) | 113 (41.9) |  |
| Middle-low | 52 (26.7) | 82 (30.4) |  |
| Middle-high | 11 (5.6) | 58 (21.5) |  |
| High | 7 (3.6) | 17 (6.3) |  |
| Alcohol intake, n (%) |  |  | 0.076 |
| Never | 116 (58.0) | 135 (49.1) |  |
| Monthly or less | 61 (30.5) | 90 (32.7) |  |
| 2-4 times per month | 12 (6.0) | 37 (13.5) |  |
| 2-3 times per week | 5 (2.5) | 7 (2.5) |  |
| 4+ times per week | 6 (3.0) | 6 (2.2) |  |
| Smoking status, n (%) |  |  | 0.059 |
| Never | 99 (49.5) | 161 (58.5) |  |
| Ex-smoker | 43 (21.5) | 59 (21.5) |  |
| Current smoker | 58 (29.0) | 55 (20.0) |  |
| Low physical activity (IPAQ)^##^, n (%) | 45 (22.5) | 70 (25.5) | 0.458 |
| Low hemoglobin, n (%) |  |  |  |
| Male <13g/dL | 18 (11.4) | 29 (11.9) | 0.869 |
| Female <12g/dL | 17 (40.5) | 3 (9.4) | 0.003* |
| Glucose, mmol/L | 4.7 (4.1-5.4) | 5.7 (5.1-7.0) | <0.001* |
| Total cholesterol (mmol/L) | 5.0 (4.5-5.5) | 4.5 (3.8-5.2) | <0.001* |
| HDL (mmol/L) | 1.3 (1.1-1.6) | 1.1 (1.0-1.4) | <0.001* |
| LDL (mmol/L) | 3.0 (2.5-3.5) | 2.4 (1.8-3.1) | <0.001* |
| eGFR <60 mL/min/m^2^, n (%) | 12 (6.0) | 50 (18.2) | <0.001* |
| GGT (U/L) | 48.0 (30.0-69.0) | 34.0 (24.0-61.0) | 0.001* |
| Albumin (g/L) | 44.0 (42.0-47.0) | 41.0 (38.5-43.0) | <0.001* |
| Hepatitis C, n (%) | 4 (2.0) | 13 (4.7) | 0.114 |
| Total CD4+ count (cells/µl) |  |  |  |
| Nadir | 109 (34-264) | 213 (49-400) | <0.001* |
| Current | 611 (439-800) | 576 (394-783) | 0.104 |
| Current CD4+ count >500 cells/ul, n (%) | 137 (68.5) | 167 (60.7) | 0.081 |
| CD4+:CD8+ ratio | 0.86 (0.62-1.20) | 0.76 (0.54-1.08) | 0.013* |
| Duration of living with HIV (years) | 13.0 (10.0-18.0) | 11.0 (5.9-17.3) | <0.001* |
| History of ADI, n (%) | 139 (69.8) | 91 (33.1) | <0.001* |
| Duration on ART (years) | 11.0 (8.0-14.0) | 10.0 (5.6-16.0) | 0.019* |
| Current ART regimen, n (%) |  |  | <0.001* |
| NNRTI-based | 160 (80.0) | 49 (17.8) |  |
| PI-based | 21 (10.5) | 33 (12.0) |  |
| INSTI-based | 16 (8.0) | 182 (66.2) |  |
| Others | 3 (1.5) | 11 (4.0) |  |
| History of treatment failure^##^, n (%) | 16 (8.0) | 20 (7.3) | 0.767 |
| Exposure to D-drugs^##^, n (%) | 131 (66.2) | 126 (45.8) | <0.001* |

Data presented in median (interquartile range, IQR), unless stated otherwise.

^#^Malaysia: household income; Hong Kong: personal income.

^##^Low physical activity is defined as < 600 metabolic equivalent (MET) - minutes per week; history of treatment failure is defined a viral load of >1000 copies/mL on two consecutive assessments while on ART; D-drugs include exposure to didanosine, stavudine, zalcitabine and zidovudine.

Abbreviations: ADI, AIDS-defining illness; ART, antiretroviral therapy; eGFR, estimated glomerular filtration rate; GGT, gamma-glutamyl transpeptidase; HDL, high-density lipoprotein; INSTI, integrase strand transfer inhibitor; LDL, low-density lipoprotein; NNRTI, non-nucleoside reverse transcriptase inhibitor; PI, protease inhibitor; PWH, people living with HIV.

^@^Chi-square or Fisher exact test performed for categorical variables and Mann-Whitney test for continuous variables.

*Significant *p*-value

Supplementary table 4: Comparison of health outcomes among PWH from the Malaysian and Hong Kong cohorts.

| **Characteristics** | **HIV+ Malaysia (n=200)** | **HIV+ Hong Kong (n=275)** | ***p*-value**^@^ |
| --- | --- | --- | --- |
| IC composite score | 5.47 (4.80-5.80) | 5.60 (4.80-5.80) | 0.220 |
| COGNITION DOMAIN | | | |
| Impaired MOCA^#^, n (%) | 50 (25.0) | 45 (16.4) | 0.020* |
| SENSORY DOMAIN (VISION AND HEARING) | | | |
| Self-reported vision impairment (VF14), n (%) | 7 (3.5) | 12 (4.4) | 0.920 |
| VF14 score | 92.9 (87.7-97.9) | 88.7 (80.0-95.2) | 0.044* |
| Self-reported hearing impairment (HHIE), n (%) | 2 (1.0) | 14 (5.1) | 0.656 |
| HHIE score | 5.0 (2.0-25.0) | 12.0 (4.0-16.0) | 0.482 |
| MOOD DOMAIN | | | |
| DASS-21, n (%) |  |  |  |
| Depression (score ≥ 7) | 32 (16.0) | 38 (13.8) | 0.508 |
| Anxiety (score ≥ 6) | 37 (18.5) | 28 (10.2) | 0.009* |
| Stress (score ≥ 10) | 13 (6.5) | 18 (6.5) | 0.984 |
| MOBILITY DOMAIN | | | |
| Slow gait speed <0.8m/s, n (%) | 19 (9.5) | 27 (9.8) | 0.908 |
| Gait speed (m/s) | 1.1 (1.0-1.2) | 1.1 (0.9-1.3) | 0.361 |
| VITALITY DOMAIN | | | |
| Nutrition status, n (%) |  |  | 0.747 |
| Normal | 153 (76.5) | 212 (77.1) |  |
| At risk of malnutrition | 43 (21.5) | 60 (21.8) |  |
| Malnourished | 4 (2.0) | 3 (1.1) |  |
| HbA1c (%) | 5.4 (5.1-5.9) | 5.9 (5.5-6.6) | <0.001* |
| Abnormal HbA1c >6.5%, n (%) | 24 (12.0) | 74 (26.9) | <0.001* |
| Low grip strength, n (%) |  |  |  |
| Men (<26kg), Women (<18kg) | 64 (32.0) | 118 (42.9) | 0.016* |
| High HsCRP ≥ 6mg/L, n (%) | 24 (12.0) | 14 (5.1) | 0.006* |
| BMI (kg/m^2^), n (%) |  |  | <0.001* |
| <18.50 (Underweight) | 20 (10.0) | 12 (4.4) |  |
| 18.50-24.99 (Normal) | 65 (32.5) | 140 (50.9) |  |
| 25-29.99 (Overweight) | 40 (20.0) | 88 (32.0) |  |
| ≥30 (Obese) | 75 (37.5) | 35 (12.7) |  |
| PRIMARY AND SECONDARY HEALTH OUTCOMES | | | |
| Frailty phenotype, n (%) |  |  | 0.307 |
| Non frail | 67 (33.5) | 85 (30.9) |  |
| Prefrail | 117 (58.5) | 176 (64.0) |  |
| Frail | 16 (8.0) | 14 (5.1) |  |
| Impaired IADL, n (%) | 55 (27.8) | 59 (21.5) | 0.113 |
| VACS index 1.0 | 12.0 (6.0-22.0) | 18.0 (10.0-37.0) | <0.001* |

Data presented in median (interquartile range, IQR), unless stated otherwise.

^#^Malaysia: demographically adjusted MOCA score, Hong Kong: cutoff ≥22 MOCA score is considered normal.

Abbreviations: BMI, body mass index; CASP-19, Control, Autonomy, Self-Realization and Pleasure quality of life scale; DASS-21, Depression Anxiety and Stress Scale 21; HHIE, Hearing Handicap Inventory for the Elderly; hsCRP, high-sensitivity C-reactive protein; IADL, Instrumental activities of daily living; IC, intrinsic capacity; MOCA, Montreal Cognitive Assessment; PWH, people living with HIV; VACS, Veterans Aging Cohort Study; VF-14, Visual Function Index; WHODAS, WHO Disability Assessment Schedule; WHOQOL-HIV-BREF, WHO Quality of Life-HIV BREF.

^@^Chi-square or Fisher exact test performed for categorical variables and Mann-Whitney test for continuous variables.

*Significant *p*-value

Supplementary figures


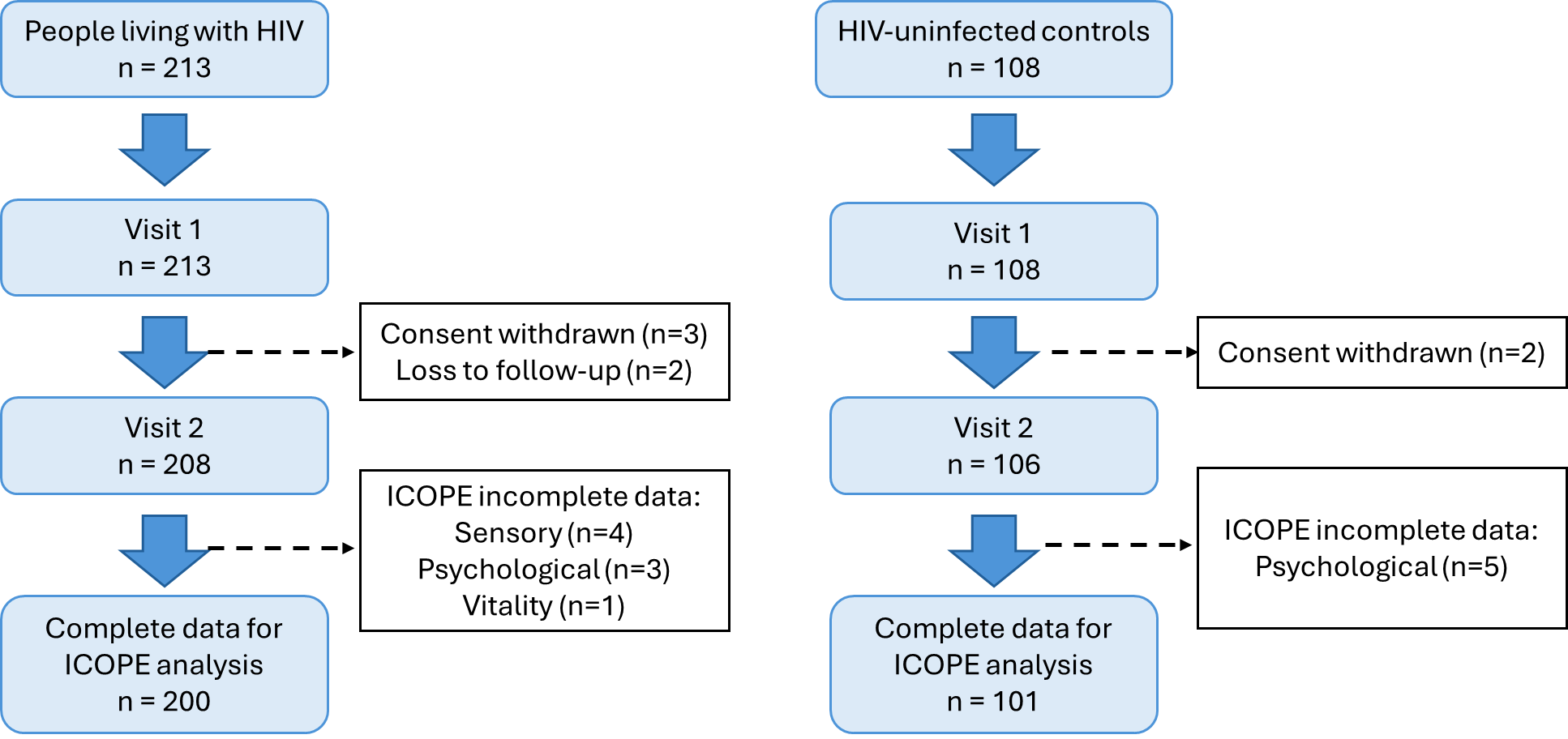


Supplementary figure 1: Flow chart of participant disposition in the Malaysian HIV and Aging cohort.


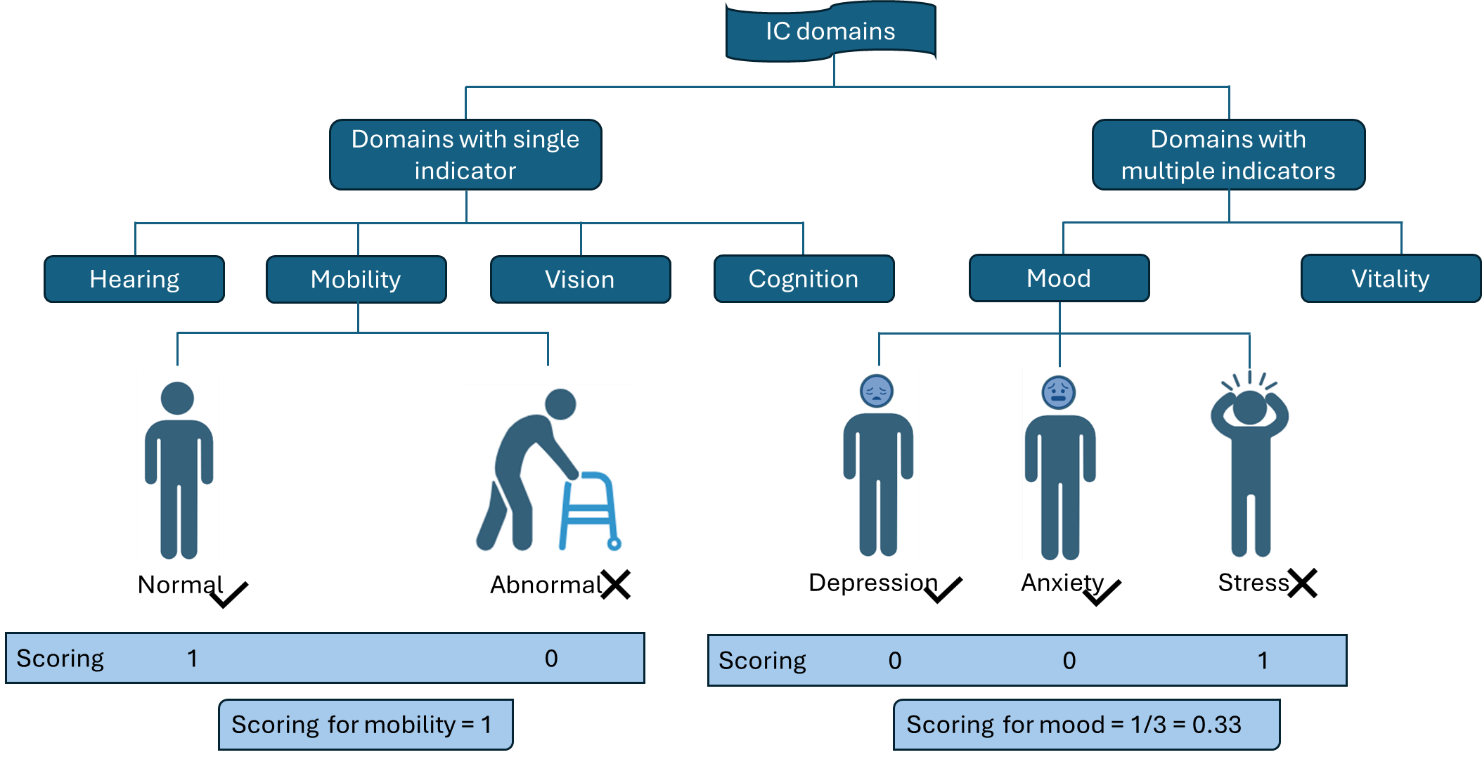


Supplementary figure 2: Illustration of how scores are calculated for intrinsic capacity domains using both single and multiple indicators. For domains with a single indicator, such as mobility, a score of 1 was given if there were no abnormalities and 0 if there was a deficit present. For domains encompassing multiple indicators, such as mood which was assessed with symptoms of depression, anxiety and stress, an average score was computed based on the number of abnormalities present. If only one symptom was present the score was 0.33 and if all symptoms present the total score was 1.


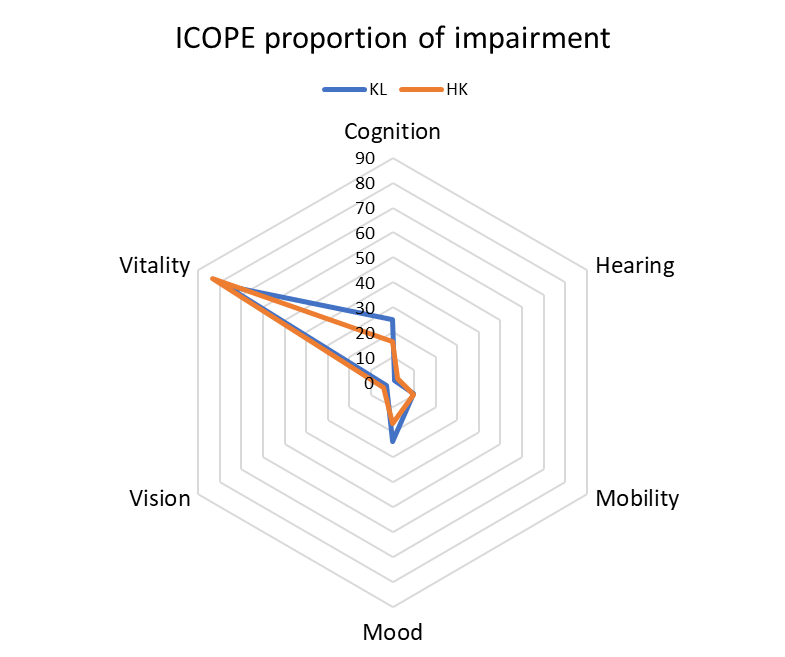


Supplementary figure 3: Radar chart comparing the frequency (%) of deficits in intrinsic capacity (IC) domains among people living with HIV (PWH) from Malaysia and Hong Kong.
